# Major depressive disorder associated alterations in the effective connectivity of the face processing network: a systematic review

**DOI:** 10.1038/s41398-024-02734-0

**Published:** 2024-01-25

**Authors:** Alec J. Jamieson, Christine A. Leonards, Christopher G. Davey, Ben J. Harrison

**Affiliations:** Department of Psychiatry, The University of Melbourne, Parkville, Victoria Australia

**Keywords:** Depression, Human behaviour

## Abstract

Major depressive disorder (MDD) is marked by altered processing of emotional stimuli, including facial expressions. Recent neuroimaging research has attempted to investigate how these stimuli alter the directional interactions between brain regions in those with MDD; however, methodological heterogeneity has made identifying consistent effects difficult. To address this, we systematically examined studies investigating MDD-associated differences present in effective connectivity during the processing of emotional facial expressions. We searched five databases: PsycINFO, EMBASE, PubMed, Scopus, and Web of Science, using a preregistered protocol (registration number: CRD42021271586). Of the 510 unique studies screened, 17 met our inclusion criteria. These studies identified that compared with healthy controls, participants with MDD demonstrated (1) reduced connectivity from the dorsolateral prefrontal cortex to the amygdala during the processing of negatively valenced expressions, and (2) increased inhibitory connectivity from the ventromedial prefrontal cortex to amygdala during the processing of happy facial expressions. Most studies investigating the amygdala and anterior cingulate cortex noted differences in their connectivity; however, the precise nature of these differences was inconsistent between studies. As such, commonalities observed across neuroimaging modalities warrant careful investigation to determine the specificity of these effects to particular subregions and emotional expressions. Future research examining longitudinal connectivity changes associated with treatment response may provide important insights into mechanisms underpinning therapeutic interventions, thus enabling more targeted treatment strategies.

## Introduction

**Fig. 1:**
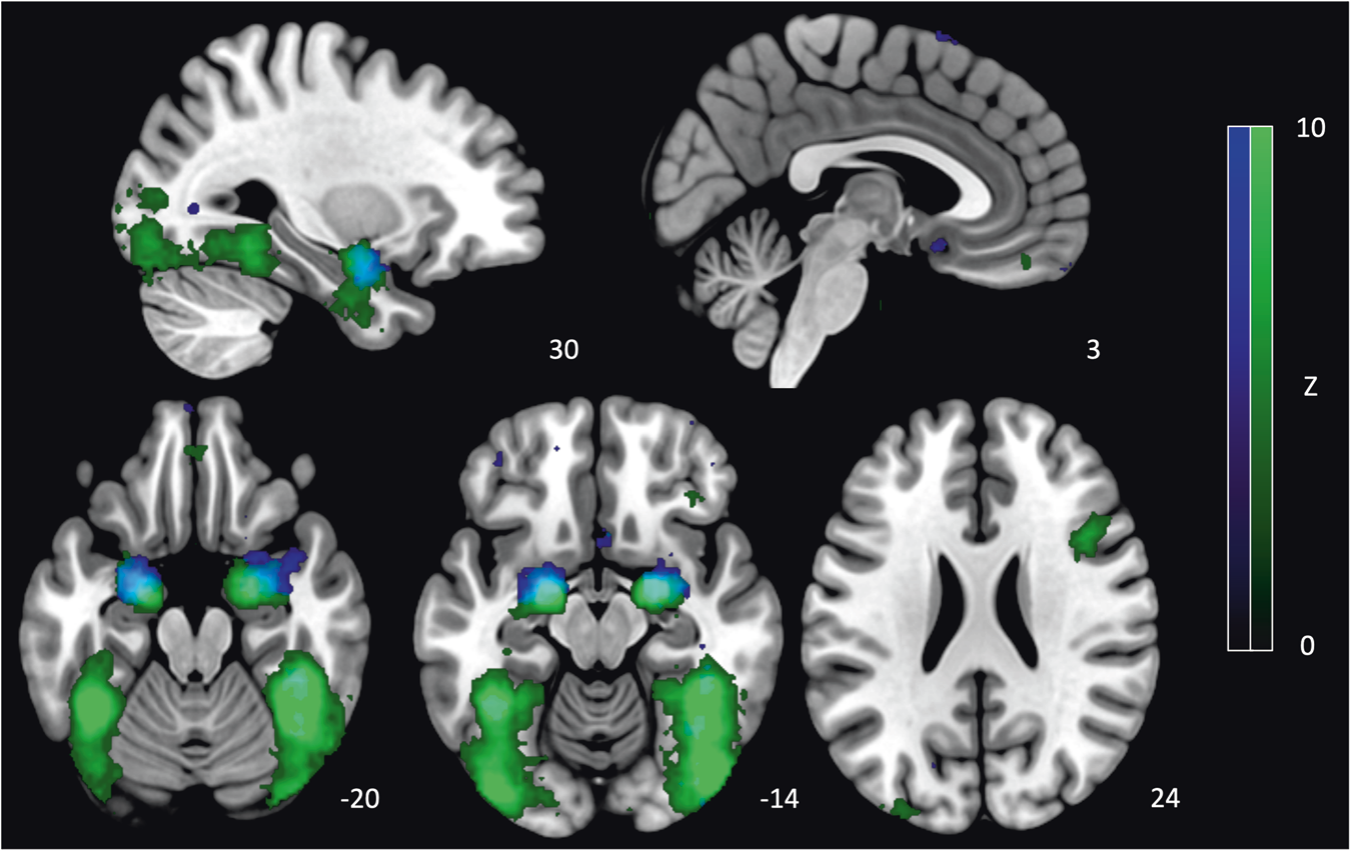
Regions consistently implicated in the processing of faces. Activation maps identified through NeuroSynth with search terms “face” (green) and “emotional faces” (blue). Color bars represent Z values.

Major depressive disorder (MDD) is a highly prevalent and disabling mental health condition, which arises due to interactions between biological, psychological, and socioeconomic factors [1–3]. Depression has been associated with an affective processing bias [4, 5], reflected by both an increased reactivity to negative emotional stimuli and a reduced reactivity to positive emotional stimuli [6]. This bias appears to be prominent in the processing of emotional facial expressions [7, 8]. The processing of emotional expressions is neurobiologically underpinned by a distributed collection of brain regions referred to as the ‘face processing network’ (see Fig. 1) [9–11]. The face processing network is commonly subdivided into core and extended subsystems, with most of the observed differences in participants with MDD occurring in the activity of the extended system [12]. Specifically, these alterations appear to predominantly occur across regions including the anterior cingulate cortex (ACC), amygdala, dorsolateral prefrontal cortex (dlPFC), and orbitofrontal cortex [12].

More recent work has sought to examine the functional connectivity between these regions and how this may also be altered in participants with MDD. Depressed participants demonstrate significantly decreased connectivity between the amygdala and dlPFC during the processing of emotional facial expressions [13, 14], particularly sad facial expressions [15]. While interactions between the dlPFC and amygdala appears to be important in the regulation of emotional responses and processing salient emotional stimuli [16–18], there are only sparse anatomical connections between these areas. Thus, it seems likely that their interactions are mediated by other regions, including parts of the ACC and prefrontal cortex [19, 20].

Distinct subregions in the ACC and ventromedial prefrontal cortex (vmPFC) appear to be associated with specific cognitive and affective functions [21–23]. Consistent with its role as a key region of the salience network, the dorsal ACC appears to be broadly involved in the integration of information in order to influence attention allocation [24]. Conversely, the subgenual ACC has been hypothesized to be a key region in regulating dysphoric emotion, due in part to its connectivity with the amygdala [25–27]. Differences between these regions may, in part, contribute to the heterogeneity in functional connectivity alterations observed in participants with MDD. For instance, depressed participants have demonstrated reduced functional connectivity between the amygdala and rostral ACC during the processing of fearful expressions [28]. Reduced connectivity between the amygdala and dorsal ACC but increased connectivity between the amygdala and subgenual ACC have also been identified [29, 30], with the former being correlated with depressive symptom severity. Evidence from other mood disorders, including bipolar disorder, indicates that depressive states differentially alter functional connectivity depending on the valence of the stimuli under investigation [31]. Versace et al. [31] found that in comparison to healthy controls and remitted bipolar disorder participants, bipolar disorder participants currently in a depressive episode illustrated increased connectivity between the amygdala and orbitofrontal cortex in response to sad expressions, but reduced connectivity from the amygdala to orbitofrontal cortex in response to happy expressions. Together, these findings suggest that the valence of the processed stimuli may also influence MDD-associated alterations to connectivity.

Although functional connectivity has been a useful method for assessing the relationship between face processing regions, it cannot infer which regions are driving these changes [32]. Effective connectivity addresses this by examining how brain regions directionally influence one another, and can be estimated using methods including dynamic causal modelling (DCM), Granger causality, and structural equation modelling. Previous reviews have not comprehensively examined MDD-associated effective connectivity during the processing of emotional expressions [12, 33]. Emotional processing paradigms remain crucial in exploring the neurobiology of MDD as they represent tasks that are well-validated and reliably activate regions commonly implicated in depression etiology [34]. Directional interactions between regions of the extended system are particularly important for informing our understanding of depression-associated alterations to emotion generation, salience processing, and emotional regulation [12, 18, 35]. As such, we aimed to provide the first systematic review of differences in effective connectivity present during the processing of facial emotions in those with MDD compared with individuals without a diagnosed mental illness. We explored whether, despite methodological differences in neuroimaging modalities and estimation methods of effective connectivity, there remained consistent findings among these studies. Furthermore, in a subgroup of studies with treatment outcome data we investigated the association between these effective connectivity parameters and changes in depressive symptoms following treatment.

## Methods

### Search criteria

This systematic review was preregistered with the International Prospective Register of Systematic Reviews (registration number: CRD42021271586) and undertaken in adherence with the Preferred Reporting Items for Systematic-Reviews and Meta-Analyses [36] (for details concerning the associated checklist see Supplementary Table S1). Systematic searches of the literature were conducted on the 16^th^ of August 2021 and the 16^th^ of May 2023, searching title and abstracts for the following keywords: “major depressive disorder” or depress* or MDD and emotion* or face or “facial expression” and “effective connectivity” or “directional connectivity” or “Granger causality” or “dynamic causal modelling” or “dynamic causal modeling” or “structural equation modelling” or “structural equation modeling”. These terms were searched through five databases which included PsycINFO, EMBASE, PubMed, Scopus, and Web of Science. The specific syntax used was adjusted across databases (see Supplementary Table S2). The inclusion criteria for these searches were: a clinical diagnosis of MDD (past or present) and a neuroimaging assessment of effective connectivity that was conducted during a task that used emotional facial stimuli. Articles were excluded if their focus was not on participants with MDD: i.e., if participants had comorbid neurological, health, or psychiatric conditions (excluding comorbid anxiety disorders). All study types, excluding reviews and case reports were included in this synthesis. Manual screening of abstracts and full texts using the aforementioned criteria was conducted by two researchers (A.J and C.L), and any discrepancies were then discussed further to determine their eligibility.

### Data extraction and synthesis

Data extraction was conducted manually by one researcher (A.J) and included the lead author, year of publication, participant characteristics (sample size, gender, age), treatment used (if applicable), measurement of depressive symptoms, neuroimaging modality and task type, assessment of effective connectivity, regions investigated (including derivation method) and key findings. The primary outcome of interest was the presence of directional interactions between brain regions and the stimuli for which they were presented. Due to differences in the selected regions, methods for assessing effective connectivity and neuroimaging modalities examined across these studies, a qualitative synthesis of the literature was undertaken rather than a meta-analytic review. For details concerning the quality assessment tools used in this review see the Supplementary Materials.

## Results

### Study characteristics

**Fig. 2:**
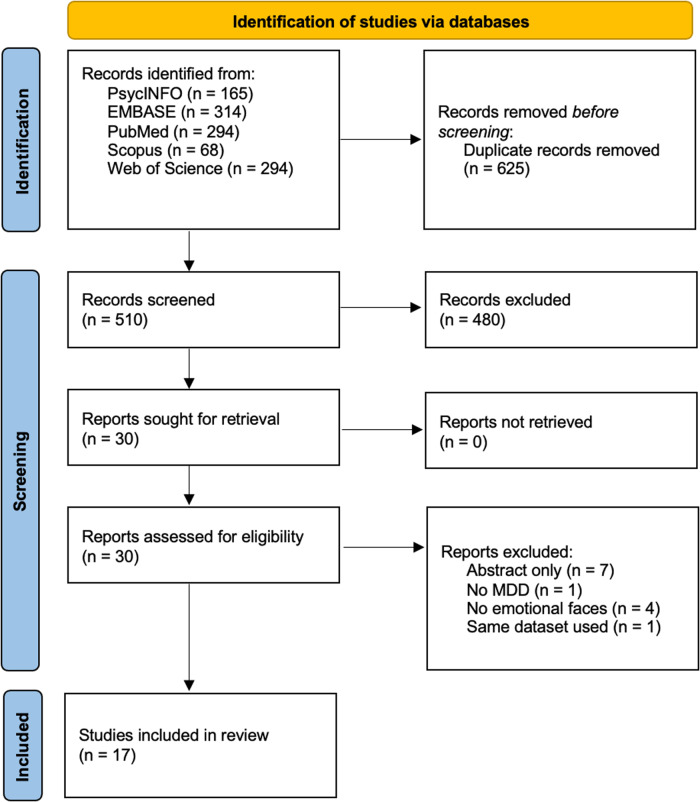
PRIMSA flow diagram detailing the filtering process for articles.

Following the removal of duplicates from the 1135 identified papers, 510 unique papers remained. Subsequent screening of titles and abstracts by the two researchers resulted in 30 papers being identified as potentially relevant to this review. Full-text screening identified a total of 17 papers that met all inclusion criteria (Fig. 2). In total, 11 studies examined effective connectivity differences in participants with MDD using fMRI [37–47], three using MEG [48–50], and three using EEG [51–53]. Thirteen of the 17 studies used DCM to assess effective connectivity [37–44, 47, 48, 50, 52, 53], three used Granger causality [45, 49, 51] and one used structural equation modelling [46]. The preference for DCM was evident in more recent studies. See Supplementary Fig. S1 for the structure of specified models from each study.

**Fig. 3:**
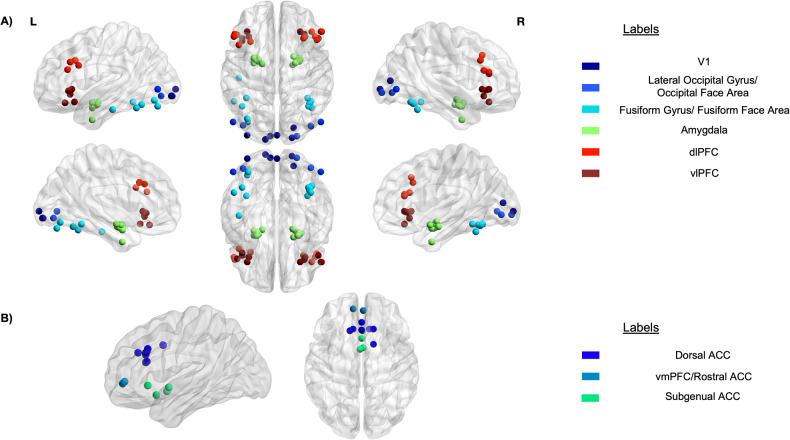
Spatial distribution of regions of interest reported across multiple studies. Center coordinates for (**A**) lateralized regions and (**B**) cortical midline structures.

By far the most investigated region was the amygdala (15 of 17 studies), followed by the fusiform gyrus (FG; 10 of 17 studies), dlPFC (seven of 17 studies), vlPFC (seven of 17 studies), dorsal ACC (seven of 17 studies), and V1 (six of 17 studies). See Fig. 3 for depiction of the distribution of regional coordinates across studies and Supplementary Fig. S2 for the percentage of studies investigating each region. Thirteen of the 17 included studies explored cross-sectional differences in effective connectivity between participants with MDD and healthy controls [37, 38, 40–43, 45–51]. Five studies examined whether baseline effective connectivity estimates could predict longitudinal changes in depressive symptoms [39, 41, 44, 50, 52]. Included studies had an average sample size of 26.9 healthy controls (range 15-89) and 31 MDD (range 5-103) participants, with only six studies having an average sample size greater than 25 for each group [39, 41–44, 47]. This is notable given that previous estimates for using frequentist comparisons in DCM suggest that for a Cohen’s *d* = 0.03, at least 27 participants are required in each group to achieve 80% power [54]. Across all included studies there was a total of 403 healthy controls and 528 MDD participants. Overall, the quality of all included studies was rated fair-to-good by both authors (for full details see Supplementary Table S3-5). Adherence to the modified fMRI reporting guidelines first conceived by Davies et al. [55] (Supplementary Table S6) was high amongst included fMRI studies (all greater than 70%; Supplementary Table S7), with an increase in adherence observed across time. Common deviations were not including details about the fMRI coil, the distance of the gap between slices in acquisition, the paradigm presentation software, the version of preprocessing and analysis software, and whether relevant covariates were adjusted for in the analysis.

With regards to behavioral analyses, seven studies reported no behavioral differences in face processing between MDD participants and healthy controls. Of the four that reported behavioral differences, one reported slower reaction time for detecting negative faces in participants with MDD compared with controls [51], one reported slower reaction times for identifying happy incongruent, sad congruent and sad incongruent trials [53], and another reported higher accuracy for MDD participants [50]. Notably, the tasks used in these studies were not the implicit or explicit face-processing tasks that are more common in the field, but a face-in-the-crowd [51], an emotional Stroop [53], and a dot-probe task [50]. The fourth study examined brain stimulation and observed a reduction in reaction time following stimulation of the subgenual ACC [52]. The remaining 6 studies did not report conducting any behavioral analyses. For full characteristics of included studies see Table 1.

**Table 1:** Summary of Studies Investigating the Effective Connectivity of Emotional Face Processing in Major Depression Disorder.

Author and year	Number of Participants (% female)	Age (SD)	Treatment Type	Assessment of Depression Severity	Imaging Modality and Emotion Processing Task Type	Effective Connectivity Method	Regions Investigated (MNI coordinates or atlas used); task-based or predefined	Main Findings
de Almeida et al. [38]	HC: 16 (75) MDD: 16 (81.2)	HC: 28.3 (8.4) MDD: 32.3 (9.7)	NA	HDRS-25 HC: NS MDD: 24.6 (6)	fMRI, event-related explicit emotional face labelling (emotional or neural) with sad and happy expressions	DCM	Bilateral vmPFC and amygdala (Wake Forest University Pick Atlas); predefined	Left hemisphere from vmPFC to amygdala was more negative in those with depression. No task associated differences in reaction time or accuracy between groups.
Carballedo et al. [46]	HC: 15 (50) MDD: 15 (50)	HC: 35.5 (10.9) MDD: 39.9 (8.6)	NA	HDRS-21 HC: NS MDD: 22.87 (4.4)	fMRI, block design explicit emotional face matching with sad and angry expressions	SEM	Bilateral amygdala (L: 28, −6, −18; R: 28, −2, −20), vlPFC (L: −34, 26, −6; R: 34, 26, −8), dorsal ACC (L: −10, 26, 30; R: 14, 26, 28), dlPFC (L: −52, 26, 32; R: 48, 22, 34); task-based	Depression-associated reduction in bilateral amygdala to vlPFC and right-side reductions from amygdala to dorsal ACC and dorsal ACC to dlPFC. No task associated differences in reaction time or accuracy between groups.
de Almeida et al. [37]	HC: 19 (63) MDD: 12 (63)	HC: 31.8 (6.8) MDD: 30.3 (7)	NA	HDRS-25 HC: 1.4 (2.2) MDD: 28.1 (6.3)	fMRI, implicit emotional face processing (coloring matching) of dynamic expressions between neural and either happy, sad, angry, or fearful	DCM	Amygdala, vmPFC, subgenual ACC (Wake Forest University Pick Atlas); predefined	Left hemisphere from vmPFC to amygdala was more negative during happy face processing in those with depression (only in females). Increased subgenual ACC to amygdala connectivity to fearful faces was also shown in depressed individuals. No task-associated differences in reaction time or accuracy between groups.
Tang et al. [51]	HC: 13 (46.2) MDD: 12 (66.7)	HC: 39.5 (9.7) MDD: 31.7 (13.6)	NA	HDRS HC: 1.85 (1.9) MDD: 27.00 (8.2)	EEG, face-in-the-crowd task using schematic faces representing displaying neural expressions with targets being either positive or negative expressions	Partial directed coherence (Granger causality)	12 electrodes: Fp1, Fp2, Fz, F5 and F6 in the prefrontal and frontal cortex, C5, C6 and Cz in the central cortex, and Pz, PO7, PO8 and Oz in the occipito-parietal cortex; predefined	Hemispheric asymmetry driven by left-frontal hypoactivity and the right-frontal hyperactivity in depressed participants. Depression was associated with slower reaction time for detecting negative faces.
Goulden et al., [40]	HC: 21 (66.7) Remitted MDD: 22 (72.7)	HC: 31.1 (10.0) Remitted MDD: 33.7 (10.7)	NA	MADRS HC: .92 (1.4) Remitted MDD: 2.31 (3.6)	fMRI, event-related implicit emotional face processing (gender matching) with happy and sad expressions	DCM	Bilateral V1 (L: -14, -98, 0; R: 18, -96, 0), FG (L: -35, -77, -20; R: 42, -60, -15), amygdala (L: -18, -7, -15: R: 25, -4, -15), and vlPFC (L: -42, 21, -15; R: 28, 32 -15); task-based	In remitted MDD happy faces modulated bidirectional FG to vlPFC connections and sad faces modulated from FG to vlPFC. Between group comparisons of parameters were not performed. No behavioral results reported.
Lu et al. [48]	HC: 20 (45) MDD: 20 (55)	HC: 31.3 (7.4) MDD: 30.7 (8.9)	NA	HDRS HC: < 8 MDD: >24	MEG, explicit recognition of sad or not from series of emotional videos displaying either eating, neutral, sad, happy and rest	DCM	Left V1 ( − 20, −84, 10), precuneus (−34, −64, 54), amygdala (−28, 0, −16), rostral ACC ( − 10, 50, −2) and dlPFC (42, 32, 18); task-based	Sad face associated modulation significantly increased connectivity from rostral ACC to dlPFC in those with depression. Intrinsic connectivity from the dlPFC to amygdala was significantly reduced, whereas amygdala to rACC was increased in those with depression. No behavioral results reported.
Lu et al. [49]	HC: 12 (100) MDD: 12 (100)	HC: 31.1 (8.6) MDD: 31.2 (7.8)	NA	HDRS HC: NS MDD: >24	MEG, explicit recognition of sad or not from series of emotional videos displaying either eating, neutral, sad, happy and rest	Granger Causality	V1, amygdala, dorsal ACC, and dlPFC (Anatomical Automated Labeling library in the Marsbar toolbox); predefined	Depressed individuals demonstrated more positive modulation from the amygdala to dorsal ACC and dlPFC around 25 and 100 ms after stimulus onset and more negative modulation from the dlPFC to amygdala about 825 ms after stimulus onset. No behavioral results reported.
Grant et al. [45]	HC: 19 (52.6) MDD: 20 (55)	HC: 31.2 (9.2) MDD: 34.5 (10.7)	NA	BDI HC: 0.79 (1.1) MDD: 31.6 (9.5)	fMRI, block design implicit emotional face processing (gender matching) with positive, sad and neutral and expressions	Granger Causality	Bilateral amygdala (L: -22, -2, -16; R: 20, 2, -16), subgenual ACC (L: -2, 2, -6; R: 2, 4, -10), midcingulate (L: -6, -8, 38), dorsal ACC (L:-14, 24, 34; R:14, 8, 38), dlPFC (L: -32, 20, 34; R: 32, 24, 40), hippocampus (Wake Forest University Pick Atlas); predefined	During the processing of negative expressions, MDD participants had reduced connectivity from the dlPFC to amygdala, midcingulate and subgenual ACC in comparison to healthy controls. MDD participants had greater connectivity from the midcingulate to amygdala, dlPFC, and subgenual ACC. No difference in the reaction time between groups.
Musgrove et al. [42]	HC: 32 (76) MDD: 27 (74)	HC: 16.1 (2.1) MDD: 15.7 (1.9)	NA	BDI HC: 2.3 (3.0) MDD: 26.4 (13.5)	fMRI, block design explicit matching of emotional faces with fearful and angry expressions	DCM	OFA (L: -41, -81, -8; R: 41, -82, -4), FG (L: -42, -58, -18; R: 40, -57, -17), amygdala (L: -26, 0, -20; R: 26, 0, -20), subgenual ACC (0, 15 -14), dorsal ACC (0, 34, 30); task-based	MDD participants had lower right hemisphere amygdala to subgenual ACC intrinsic connectivity. No difference was found in modulation (combination of fearful and angry expressions). No behavioral results reported.
Vai et al. [44]	HC: 31 (58.1) MDD: 33 (57.6) [Treatment Remitter: 21 (52.4) Treatment Non-remitter: 12 (66.7)]	HC: 30.3 (10.0) MDD: 30.3 (11.2) [Treatment Remitter: 30.1 (11.8) Treatment Non-remitter: 30.9 (10.1)]	Escitalopram 10 mg for 5/6 weeks	HDRS HC: 0.4 (.8) MDD: 23.0 (4.5) [Treatment Remitter: 22.8 (4.9) Treatment Non-remitter: 23.3 (4.0)]	fMRI, block design implicit emotional face processing (gender matching) with fearful and happy expressions	DCM	FG, amygdala, dorsal ACC, vlPFC (Wake Forest PickAtlas software); predefined	Connectivity from the dorsal ACC to amygdala was greater in treatment non-remitters compared with healthy controls during fearful face processing. Intrinsic connectivity from the dorsal ACC to amygdala and amygdala to vlPFC was greater in non-remitters compared to controls. Treatment responders and HC did not significantly differ in any connectivity parameters. No behavioral results reported.
Kibleur et al. [52]	MDD: 5 (80)	MDD: 52 (4.1)	Deep brain stimulation to the subgenual ACC (within participant repeated measures on or off for different conditions in the task)	HDRS-17 MDD: 23.8 (4.1)	EEG, event related emotional Stroop task with neutral, fearful, and happy expressions	DCM	V1 (0, −93, 0), bilateral LOC ( ± 53, −77, 0), FG ( ± 37, −48, −15) PCC (0, −46, 27), temporal poles (±25, 0, −36), dorsal ACC (0, 25, 31) and vlPFC (±50, 24, 0); task-based	Subgenual ACC stimulation reduced connectivity to and from the temporal poles and was associated with a reduction in depressive symptoms. Brain stimulation was associated with a reduction in RT.
Frässle et al. [39]	Treatment Remitter: 39 (72) Treatment Responder: 31 (65) Treatment Non-responder: 15 (60)	Treatment Remitter: 35.9 (11.5) Treatment Responder: 35.0 (10.0) Treatment Non-responder: 44.0 (10.0)	Non-standardized treatment (naturalistic setting)	Inventory of depressive symptomatology Treatment remitter: 31.44 (10.8) Treatment responder: 32.8 (8.4) Treatment Non-remitter: 33.72 (7.9)	fMRI, event-related emotional implicit emotional face processing (gender matching) with happy, sad, fearful, and angry faces	DCM	Bilateral OFA, FG, amygdala (Neurosyth “face”); predefined	Right amygdala to FG connectivity during happy face processing influences remission the most. This amygdala to FG connectivity was more inhibitory in remitters compared with non-remitters, however, none of the highlighted differences survived the multiple comparisons correction. No behavioral results reported.
Gilbert et al. [50]	HC: 15 (73.3) MDD: 19 (57.9)	HC: 34.7 (11.8) MDD: 36.7 (10.9)	Ketamine hydrochloride 0.5 mg/kg, single dose and saline placebo	MADRS HC: 1.5 (1.6) MDD: 33.4 (4.7) (from full sample)	MEG, dot-probe task with an emotional (happy or angry) and neutral expression (required to indicate which side dot was presented following the display of the faces)	DCM	Left VI ( − 8, −94, −8), FG (−52, −52, −22), amygdala (−25, −3, −16), vlPFC (−48, 28, −2); task-defined	Treatment with ketamine was associated with increased NMDA transmission in the FG, and slower NMDA transmission in the amygdala. Reduction of depressive symptoms following treatment was associated with AMPA transmission in the early visual cortex. No reaction time differences, however, the MDD group was more accurate than controls.
Jamieson et al. [41]	HC: 89 (56.5) MDD: 77 (61.4) [Treatment Responders: 37 (62.2) Treatment Non-responders: 40 (67.5)]	HC: 20.1 (2.9) MDD: 19.8 (2.7) [Treatment Responders: 19.6 (2.8) Treatment Non-responders: 20.0 (2.6)]	12 weeks of CBT and either a placebo or a daily 20-mg capsule of fluoxetine	MADRS HC: 2.1 (2.8) MDD: 32.4 (7.1) [Treatment Responders: 31.95 (5.1) Treatment Non-responders: 33.28 (5.9)]	fMRI, block design implicit emotional face processing (gender matching) with sad and fearful expressions	DCM	HC: Right OFA (28, -92, -8), FG (40, -60, -18), amygdala (20, -6, -16), dlPFC (50, 26, 20), and vmPFC (0, 46, -2); task-defined MDD: Right OFA (24, -94, -4), FG (38, -58, -16), amygdala (22, -6, -12), dlPFC (46, 26, 22), and vmPFC (2, 48, 0); task-defined	Depression-associated with reduced inhibition from the dlPFC to vmPFC and reduced excitation from the dlPFC to amygdala during sad expression processing. Treatment response was associated with connectivity from the amygdala to dlPFC during sad expression processing and amygdala to vmPFC connectivity during fearful expression processing. No behavioral difference in reaction time or accuracy.
Sacu et al. [43]	HC: 48 (70.8) MDD-First Degree Relative: 49 (67.3) MDD: 103 (61.1)	HC: 31.3 (9.3) MDD- First Degree Relative: 28.5 (8.1) MDD: 31.9 (9.0)	NA	BDI HC: 1 (3.5) MDD- First Degree Relative: 3 (4.5) MDD: 21 (16.5)	fMRI, block design implicit emotional face processing (identity matching) with angry and fearful expressions	DCM	Bilateral FG (L: -36, -73, -13; R: 39, -55, -16), amygdala (L: -24, -4, -19; R: 27, -4, -19), dorsal ACC (9, 26, 20), dlPFC (L: -51, 29, 23; R: 54, 32 20), vlPFC (L: -36, 32, -16; R: 33, 32, -16) and insular (L: -36, 23 -1; R: 36, 29, -1); task-defined	Depression was associated with greater negative effective connectivity from the left amygdala and left dlPFC to the right FG as well as from the left vlPFC to left FG. No behavioral differences in reaction time or accuracy.
Willinger et al. [47]	HC: 33 (70) MDD: 30 (67)	HC: 16.2 (1.9) MDD: 16.1 (1.4)	NA	CDI HC: 8.4 (6.6) MDD: 29.6 (9.3)	fMRI, block design implicit emotional face processing (gender matching) with dynamic positive, negative and neutral valenced expressions	DCM	Right FG (41, -52, -24), amygdala (19, -8, -18), vlPFC (53, 32, 0) and subgenual ACC (1, 24, -4); task-defined	Depressed participants illustrated decreased connectivity from the subgenual ACC to vlPFC, subgenual ACC to amygdala as well as increased connectivity from the vlPFC to subgenual ACC. No behavioral differences in reaction time or accuracy.
Li et al. [53]	HC: 20 (55) MDD: 20 (45)	HC: 37.3 (11.3) MDD: 31.9 (9.4)	NA	PHQ-9 HC: 0.7 (1.2) MDD 17.8 (5.3)	EEG, event related emotional Stroop task with sad and happy expressions (only examined happy expressions for DCM analysis)	DCM	Left V1 (-36, 93, -8), inferior temporal gyrus (-14, -16, -48), FG (-46, -24, -24), hippocampus (-28, -78, 16), amygdala (-24, 0, -22), and dlPFC (-46, 16, 28); predefined and task-defined	In MDD participants, incongruent happy faces modulated connectivity from the FG to amygdala and inferior temporal gyrus as well as from the amygdala to inferior temporal gyrus. Between group comparisons of paramters were not performed. Reaction time was slower for MDD participants for happy incongruent, sad congruent and sad incongruent trials.

*ACC* anterior cingulate cortex, *BDI* Beck Depression inventory, *CBT* cognitive behavioral therapy, *CDI* Children Depression Inventory, *DCM* Dynamic causal modelling, *dlPFC* dorsolateral prefrontal cortex, *EEG* electroencephalogram; *FG* fusiform gyrus, *fMRI* functional magnetic resonance imaging, *HC* healthy controls, *HDRS* Hamilton Depression Rating Scale, *NA* not applicable, *NS* not stated, *MDD* Major Depressive Disorder, *MEG* Magnetoencephalography, *MNI* Montreal Neurological Institute, *OFA* occipital face area, *PHQ-9* Patient Health Questionnaire, *SD* standard deviation, *SEM* structural equation modelling, *V1* primary visual cortex, *vlPFC* ventrolateral prefrontal cortex, *vmPFC* ventromedial prefrontal cortex.

### Systematic review findings

**Fig. 4:**
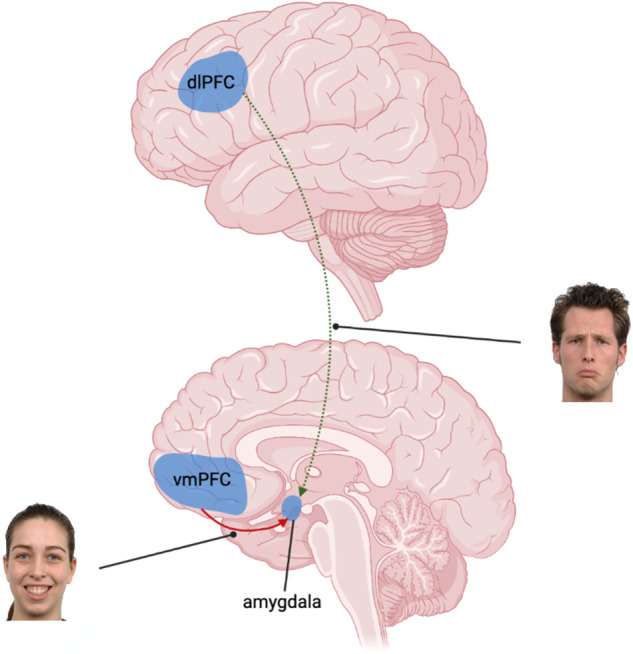
Diagram illustrating findings found across multiple studies relating to the processing of positively and negatively valenced expressions. Red solid line indicates greater inhibitory connectivity, green dotted line indicates reduced connectivity (reduced excitatory or greater inhibitory connectivity) for the MDD group. Image created with BioRender (www.biorender.com).

The most consistent finding was that participants with MDD had reduced connectivity from the dlPFC to the amygdala during the processing of negatively valenced stimuli in comparison to healthy controls (Fig. 4) [41, 45, 49]. Relatedly, one study also observed significantly reduced intrinsic connectivity from the dlPFC to the amygdala in those with depression [48]. As such, four of the seven studies examining the dlPFC and amygdala illustrated this ‘top-down’ alteration, including 67% of implicit and explicit face processing tasks examining these regions. This difference was observed across estimation methods for assessing effective connectivity, including Granger causality and DCM, as well as in fMRI and MEG research. While this reduction appears to be generally related to negatively valenced emotional expressions, one study has suggested it may be specific to sad facial expressions and not present during the processing of fearful expressions [41].

Two studies further reported greater inhibitory connectivity from the left vmPFC to amygdala during the processing of happy facial expressions in MDD participants compared with controls (Fig. 4) [37, 38]. Greater inhibition appears specific to positively valenced expressions, as other work examining this connection during the processing of fearful faces has revealed reduced inhibitory connectivity from the vmPFC to amygdala [41]. These two papers represented 50% of the studies investigating the vmPFC/rACC and 67% of those investigating these regions and happy facial expressions. Nearby regions, including the subgenual ACC have shown conflicting evidence, with some research demonstrating lower amygdala to subgenual ACC intrinsic connectivity [42], lower subgenual ACC to amygdala intrinsic connectivity [47], and increased subgenual ACC to amygdala connectivity during fearful face processing in MDD participants [37].

While most of the studies compared differences between MDD participants and controls, five of 17 examined associations between connectivity and treatment response [39, 41, 44, 50, 52]. Due to heterogeneity in the treatments and regions examined, there were no directly replicated findings across studies. Interactions between the FG and amygdala, however, appear to be broadly implicated across multiple studies. Baseline modulation from the amygdala to FG was more inhibitory for remitters compared with non-remitters during the processing of happy faces [39]. Relatedly, stimulation of the subgenual ACC was associated with reduced excitatory connectivity from the right temporal pole to the right FG [52], with this temporal pole area including the amygdala. Gilbert et al. [50] found faster NMDA transmission in the FG and slower NMDA transmission in the amygdala in those with treatment-resistant depression following ketamine treatment. However, these changes were not associated with reductions in depressive symptom severity [50].

## Discussion

Our systematic review of effective connectivity alterations in MDD during the processing of emotional facial expressions has confirmed three major findings. First, MDD is marked by a reduction in effective connectivity from the dlPFC to amygdala during the processing of negatively valenced facial expressions. Second, there is greater inhibitory connectivity from the left vmPFC to amygdala during the processing of happy facial expressions in MDD participants. And third, there is emerging evidence of an association between treatment response and interactions from the amygdala to FG.

### Connectivity from the dlPFC to amygdala

The reduced directed connectivity from the dlPFC to the amygdala during the processing of negatively valenced stimuli [41, 45, 49] is consistent with findings from functional connectivity studies demonstrating reduced amygdala and dlPFC connectivity in participants with MDD [13]. Compared with healthy controls, MDD participants have been shown to demonstrate increased amygdala reactivity for emotional tasks as well as decreased dlPFC activity during cognitively demanding tasks [56, 57]. In addition to emotional processing tasks, reduced connectivity between these regions has been observed for MDD participants in resting-state functional connectivity analyses [58, 59], with increased connectivity following treatment being associated with greater depressive symptom score reductions [60]. Given that connectivity between the amygdala and dlPFC has been associated with one’s ability to downregulate negative emotions [61], depression-associated changes to this connectivity may underpin the altered regulation of emotional responses common to this disorder [16, 18].

The dlPFC is typically recruited during voluntary and explicit emotional regulation, and thus for healthy individuals, the dlPFC has a less prominent role in the implicit emotional regulation that occurs during the processing of emotional stimuli [62]. Altered recruitment of the dlPFC during automatic cognitive control tasks in those with MDD might arise in an attempt to overcome reactivity of the amygdala induced by emotional contexts [63]. In turn, this may be due to insufficient regulation occurring from regions commonly involved in implicit regulation. Findings from the effective connectivity literature further support this interpretation by consistently demonstrating that these alterations originate in the dlPFC, rather than the amygdala. Notably, due to inconsistencies between studies, including task design, participant age, and how the connectivity parameters were modelled (e.g., mean-centering of the direct input), it is unclear whether this effect represents greater inhibitory or reduced excitatory connectivity in MDD participants in comparison to healthy controls. A greater variety of studies examining these regions will hopefully clarify which of these factors may be modifying this baseline connectivity in healthy individuals.

It is also likely given the sparse anatomical connectivity between the amygdala and dlPFC that their interactions are mediated by other regions, including the dorsal, rostral, and subgenual ACC [19]. Connectivity differences between the amygdala and ACC were noted in the majority (5 of 7) of studies investigating these regions [38, 42, 46, 48, 64], despite limited consistency concerning the directionality (from the ACC to the amygdala or the amygdala to ACC) and direction (MDD participants demonstrating greater connectivity than healthy participants or vice versa) of this effect. This is important as these regions, in addition to the medial prefrontal cortex, have been suggested to be involved in recruiting the dlPFC to produce appropriate automatic emotional regulation in those with MDD [63]. Reduced connectivity has also been observed from the dlPFC to sgACC [45], whereas greater connectivity has been found from the dlPFC to vmPFC [41] and from the rostral ACC to dlPFC during the processing of sad expressions [48]. Resting-state effective connectivity studies further highlight the centrality of rACC dysfunction in MDD [65], with greater negative connectivity being illustrated from this region to the bilateral dlPFC, insular, dACC and left amygdala. Nevertheless, it remains unclear from these findings whether any one region or a combination of regions are responsible for mediating the relationship from the dlPFC to amygdala.

In addition to these depression-associated differences, the hypothesized function of the ACC during emotional regulation may contribute to the relevance of such regions in predicting treatment response [66–69]. For example, Godlewska et al. [70] illustrated that in participants with MDD, dorsal ACC activity to sad compared with happy masked faces was predictive of treatment response to escitalopram. Other effective connectivity research has similarly shown that baseline connectivity between the dorsal ACC to amygdala was greater in MDD non-responders compared with healthy controls during fearful face processing [44]. As such, consideration of cingulate connectivity in conjunction with the interactions between the amygdala and dlPFC may provide a more accurate model of this pathway. The development of more precise models may, in turn, have greater utility in predicting response to treatment.

### Connectivity from the vmPFC to amygdala

Although two studies demonstrated greater negative connectivity from the left vmPFC to amygdala during the processing of happy facial expressions [37, 38], in one study this was observed in a secondary analysis conducted only in female participants [37]. Interestingly, the interaction from the vmPFC to amygdala has also been shown to be reduced during the processing of fearful expressions [41], an effect that has also been observed in functional connectivity studies [28]. This suggests that the valence of the stimuli under investigation may influence these results. While this has not yet been directly examined in participants with MDD, it would be consistent with findings from healthy controls demonstrating the medial and vmPFC’s sensitivity to valence [64, 71]. Notably, both positively and negatively valenced expressions were directly examined in a recent study by Willinger et al. [47]; however, modulation-specific effects were not observed between interactions of the amygdala, subgenual ACC, and vlPFC.

In contrast to the dlPFC, the vmPFC is more commonly associated with implicit regulation, which is evoked automatically and without insight [18]. Importantly, while the proximal vlPFC has commonly been viewed as a region involved in explicit regulation, there is also evidence for its role in implicit emotional regulation [72, 73]. The vlPFC is also a potential mediator between the dlPFC and vmPFC in the regulation of amygdala activity [74–76]. This relationship is likely influenced by the valence of the stimuli, as participants with MDD demonstrate reduced dlPFC and vlPFC functional connectivity during the processing of happy facial expressions [77]; however, this connectivity has been observed to increase for sad and angry facial expressions [78]. While there have been several investigations of vlPFC effective connectivity [40, 43, 44, 46, 47, 50, 52], this has often not been conducted in consideration of other prefrontal regions. Thus, comprehensive examination of the vlPFC, vmPFC, amygdala and dlPFC together in future studies may enable a more complete understanding of depression-associated dysfunction between prefrontal and subcortical circuitry.

### Connectivity from the amygdala to fusiform gyrus

The broader literature provides insights into the potential significance of amygdala to FG connectivity in predicting response to treatment. While there is limited direct evidence from effective connectivity studies of depression, alterations in amygdala and FG connectivity have been shown in functional connectivity studies. For instance, during an implicit negative face matching task functional connectivity between the amygdala and FG has been shown to be reduced in participants with MDD and their relatives compared to controls [14]. These findings are broadly consistent with results of greater negative connectivity from the left amygdala to right FG [43]. Notably, the analyses conducted in Wackerhagen et al. [14] and Sacu et al. [43] were undertaken in the same participant sample and thus are not independent sources of evidence. Examination of remitted MDD participants also reveals more negative functional connectivity between the amygdala and FG during the processing of sad expressions and more positive connectivity during the processing of happy expressions in comparison with healthy controls [40]. This finding is supported by a meta-analysis of fMRI tasks examining emotional stimuli which showed valence-dependent effects in the activation of both the amygdala and FG in participants with depression [79]. Feedback from the amygdala has been suggested to play an important role in the optimization of visual information encoding to better enable the prediction of aversive events [80]. Indeed, repeated exposure to the same stimuli appears to be associated with reduced connectivity from the amygdala to FG [81]. As such, this may represent a more general-purpose function in which the amygdala influences the prioritization of the processing of stimuli of high social or emotional relevance [82]. The changes to this connectivity demonstrated in participants with MDD would, therefore, represent alterations to the prioritization of positively and negatively valenced expressions by the early visual system, which, in turn, occurs as a result of altered feedback of information from prefrontal regions. Greater negative connectivity in this pathway may represent a more typical form of depression which has greater sensitivity to commonly used treatments [39]. Moreover, this interpretation is supported by the finding that stimulation of the subgenual ACC results in both downstream reductions to this connectivity and reductions in depressive symptoms [52]. This highlights the importance of modelling both the core and extended face processing networks despite the notion that depression-associated effects are localized to the areas of the extended system.

### Quality of the included studies

As illustrated through the quality assessment tools, all studies were rated by the authors as having either a ‘fair’ or ‘good’ quality and were consistent with reporting guidelines. However, there remain several limitations and considerations which were consistent among multiple studies. First, there was a moderate amount of variation across studies in terms of the task type, the regions of interest, the stimuli used, and the treatments applied. All these factors are likely to add to the heterogeneity in results, which have been observed and pose further difficulties in disentangling “true effects” due to the small number of studies in the area. It is also worth noting that despite the amygdala being implicated in all of the results highlighted above, this may be a function of the number of studies that included this as a region of interest. Only two studies did not explicitly examine this region, though one of these attempted to examine it indirectly [52]. This is likely to bias the overall findings away from implicated regions that were less frequently examined as regions of interest, including the insular, which despite its importance to emotion processing was only examined in one study [43]. Finally, like many neuroimaging studies, much of the work highlighted in this review had relatively few participants [83]. This is likely to result in low statistical power and increased risk of false positives, particularly in studies that conducted subgroup analyses. None of the included studies performed an a priori power analysis to determine whether they had a sufficient sample size. These issues are particularly important for interventional studies and those which aim to have clinical relevance, as underpowered studies result in claims that lack generalizability beyond the sample under investigation [83, 84]. While data suggests that methodologies, including measures of effective connectivity, may aid in reducing the number of participants required to detect between-group effects [54], the precise numbers required are likely to vary due to the task and parameters in question. If, as suggested by previous research, alterations to these interactions relate to specific symptoms or symptom profiles rather than MDD as a whole [85, 86], much larger sample sizes than those shown in this review will be necessary to accurately examine these relationships.

### Future directions

Most studies have focused on the analysis of connectivity changes during the processing of negatively valanced facial expressions. In contrast, few studies examined positively valenced expressions, which made determining which effects may be valence-specific difficult. Examination of the interactions highlighted above with a wider range of emotional expressions would aid in disentangling any valence specific effects. Further, there remains a paucity of studies examining the association between treatment response and these directional interactions. Potentially more importantly when considering the potential mechanisms of therapeutic interventions, no studies have examined longitudinal changes in effective connectivity following treatment. Determining the reliability of these estimates within depressed participants is an important step in illustrating the usefulness of identified connectivity parameters as biomarkers. Through the use of 7 Tesla imaging, more accurate examination of the effective connectivity of subcortical regions, which were largely unexamined in this review, may additionally be achieved. The effective connectivity of these regions, particularly the thalamus, have received recent attention for their importance in MDD-associated alterations at rest [87] and their association with repetitive negative thinking in healthy controls [88].

Consideration of potential confounders is also a necessary step going forward, particularly in terms of the effects of age and gender as these factors are known to influence MDD presentation [89–92]. Recent advancements in the methodology for assessing between-group differences and the effects of covariates, including parametric empirical Bayes for DCM [93], will be important tools in minimizing these effects in future studies.

## Conclusions

While investigations into depression-associated alterations in effective connectivity during the processing of emotional faces have been occurring for over a decade, only recently have enough studies been published to determine consistent findings. This early work has illustrated direct evidence for altered connectivity from the prefrontal cortex to the amygdala, which is suggested to vary according to the valence of the stimuli. Overall, the findings from this review provide a framework for future research to further disentangle the specificity and generalizability of the associations highlighted above. Additional work examining the predictive validity and clinical utility of these connectivity parameters longitudinaly will aid in providing a more nuanced understanding of the neurobiological mechanisms underlying treatment response in depression.

### Supplementary information

Supplementary Materials

## Data Availability

Not applicable.
