# Supplementary material for: Major depressive disorder associated alterations in the effective connectivity of the face processing network: a systematic review
Source: Transl Psychiatry. 2024 Jan 25;14:62. doi: 10.1038/s41398-024-02734-0 (PMC10810788; doi:10.1038/s41398-024-02734-0)
Supplement: Supplementary file 1 — Supplementary Materials [file 41398_2024_2734_MOESM1_ESM.docx]

**Supplementary Materials**

**Supplementary Methods……………………………………………………………..………2**

**Supplementary Figures……………………………………………………………..………..3**

Supplementary Figure S1: Model structures specified in each of the included studies….……3

Supplementary Table S2: Percentage of studies which investigated given brain regions for areas which appeared in more than two studies ………………………..……..……………....4

**Supplementary Tables…………………………………………………………………….…5**

Supplementary Table S1: Preferred Reporting Items for Systematic Reviews and Meta-Analyses Checklist.………..………………………………………………..………………....5

Supplementary Table S2: Specific Search Strings Used for PsycINFO, EMBASE, PubMed, Scopus, and Web of Science and the Associated Number of Search Results.…………..….....9

Supplementary Table S3: Quality Assessment Tool for Observational Cohort and Cross-Sectional Studies…………..………………………………………..……..………………....11

Supplementary Table S4: Quality Assessment of Controlled Intervention Studies….……...13

Supplementary Table S5: Quality Assessment for Before-After (Pre-Post) Studies with No Control Group………………………………………………………………………………..14

Supplementary Table S6: MRI Research Quality Assessment Tool Based on the Committee on Best Practices in Data Analysis and Sharing Guidelines…….……………………….…..15

Supplementary Table S7: MRI Research Quality Assessment Tool……………….………..16

**Supplementary References……………………………………………………….……..….17**

**Supplementary Methods**

**Quality Assessment**

Quality assessments for included studies were independently assessed by two researchers (A.J and C.L), using the National Heart, Lung and Blood Institute’s quality assessment tools ^1^. These are a set of questionnaires that aid in assessing potential methodological flaws and highlighting details that are crucial for internal validity in a manner that is comparable between studies. Based on their adherence to the assessment tools, each study was rated either “Poor”, “Fair” or “Good”. For cross-sectional studies, questions 6, 7, 10 and 13 were removed from the Quality Assessment Tool for Observational Cohort and Cross-Sectional Studies due to their lack of applicability for the studies under investigation. See the notes for Supplementary Table S3-5 for a list of the questions used in these assessments.

In addition to this general assessment, we also assessed the included fMRI studies on a modified version of the specialist protocol for MRI research quality devised by Davies et al. ^2^ (Supplementary Table S6). This assessment tool was based on the published guidelines on how to report imaging research by the Organization of Human Brain Mapping’s Committee on Best Practices in Data Analysis and Sharing ^3^. A maximum score of 20 is available if relevant sections were correctly reported in the manuscript (range 0-20).


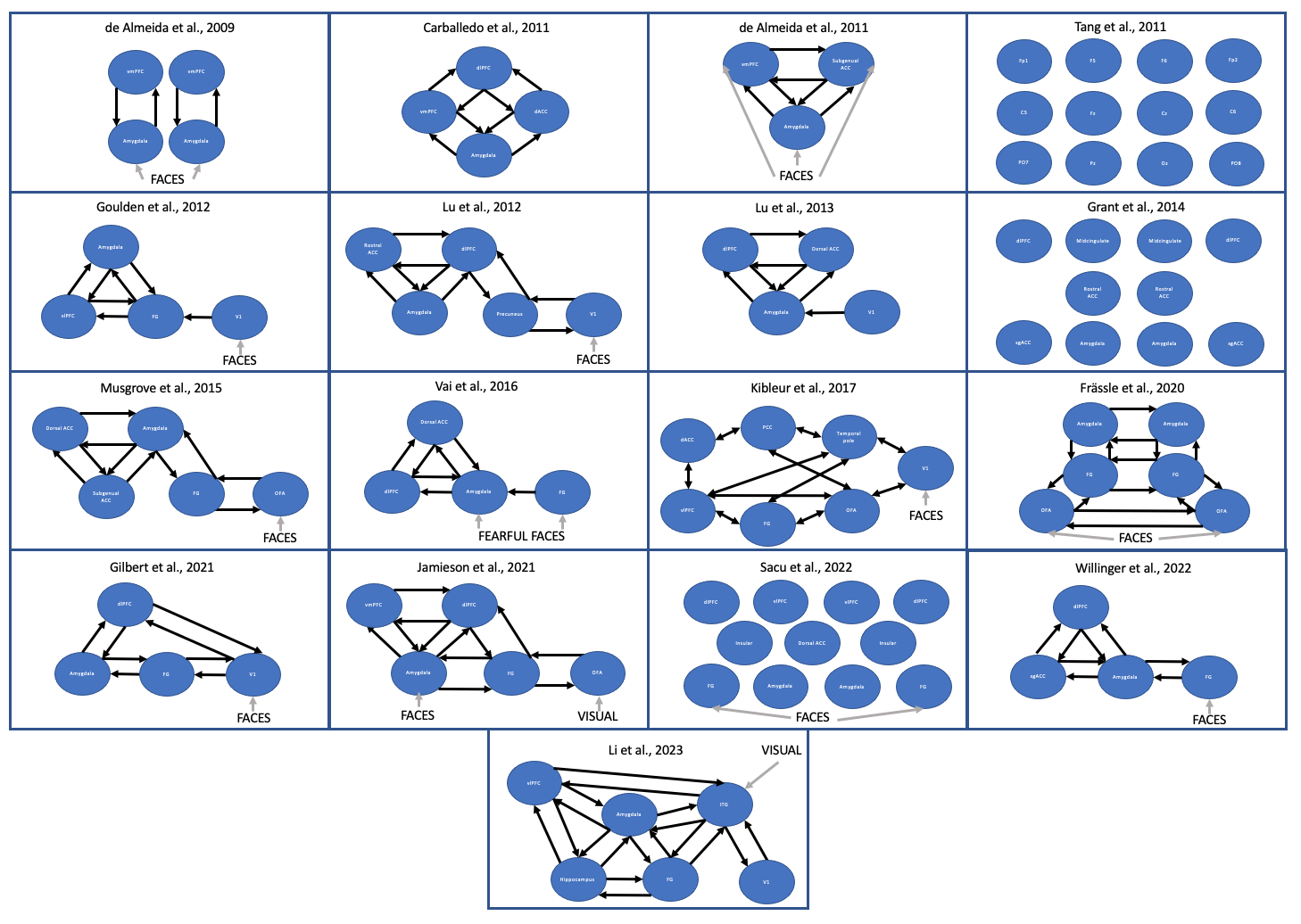


*Supplementary Figure S1.* Model structures specified in each of the included studies. Intrinsic connections are shown in black, driving inputs are depicted in grey. For models which are depicted with no intrinsic connections, all regions were connected to all other regions.

*Abbreviations:* ACC = anterior cingulate cortex; dlPFC = dorsal lateral prefrontal cortex; FG = fusiform gyrus; OFA = occipital face area; V1 = primary visual cortex; vlPFC = ventrolateral prefrontal cortex; vmPFC = ventromedial prefrontal cortex


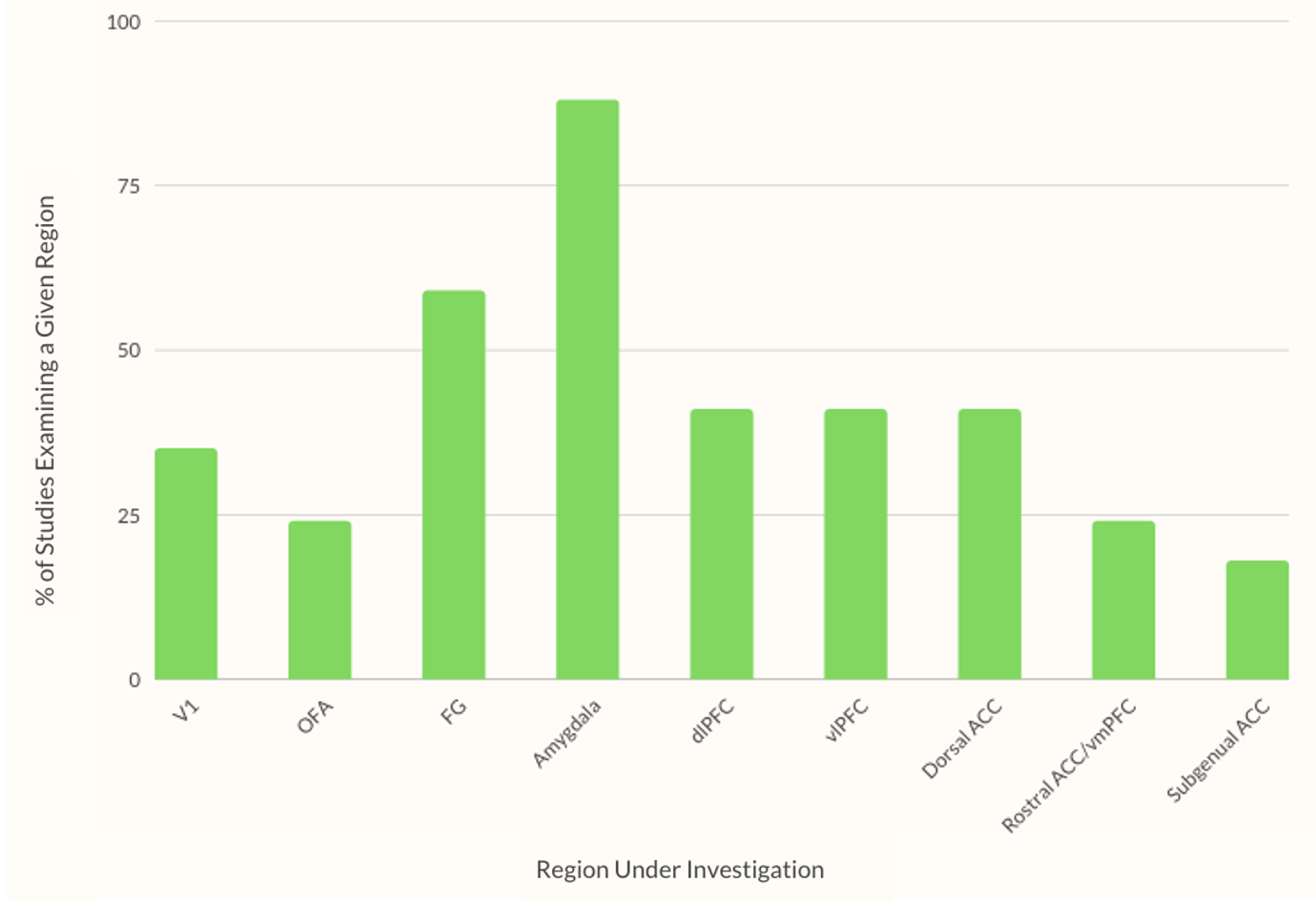


*Supplementary Figure S2.* Percentage of studies which investigated given brain regions for areas which appeared in more than two studies.

*Abbreviations:* ACC = anterior cingulate cortex; dlPFC = dorsal lateral prefrontal cortex; FG = fusiform gyrus; OFA = occipital face area; V1 = primary visual cortex; vlPFC = ventrolateral prefrontal cortex; vmPFC = ventromedial prefrontal cortex

Supplementary Table S1

### *Preferred Reporting Items for Systematic Reviews and Meta-Analyses Checklist.*

| **Section and Topic** | **Item #** | **Checklist item** | **Location where item is reported** |
| --- | --- | --- | --- |
| **TITLE** | | |  |
| Title | 1 | Identify the report as a systematic review. | 1 |
| **ABSTRACT** | | |  |
| Abstract | 2 | See the PRISMA 2020 for Abstracts checklist. | 2 |
| **INTRODUCTION** | | |  |
| Rationale | 3 | Describe the rationale for the review in the context of existing knowledge. | 3-4 |
| Objectives | 4 | Provide an explicit statement of the objective(s) or question(s) the review addresses. | 4 |
| **METHODS** | | |  |
| Eligibility criteria | 5 | Specify the inclusion and exclusion criteria for the review and how studies were grouped for the syntheses. | 5 |
| Information sources | 6 | Specify all databases, registers, websites, organisations, reference lists and other sources searched or consulted to identify studies. Specify the date when each source was last searched or consulted. | 5 |
| Search strategy | 7 | Present the full search strategies for all databases, registers and websites, including any filters and limits used. | Table S2 |
| Selection process | 8 | Specify the methods used to decide whether a study met the inclusion criteria of the review, including how many reviewers screened each record and each report retrieved, whether they worked independently, and if applicable, details of automation tools used in the process. | 5 |
| Data collection process | 9 | Specify the methods used to collect data from reports, including how many reviewers collected data from each report, whether they worked independently, any processes for obtaining or confirming data from study investigators, and if applicable, details of automation tools used in the process. | 5 |
| Data items | 10a | List and define all outcomes for which data were sought. Specify whether all results that were compatible with each outcome domain in each study were sought (e.g. for all measures, time points, analyses), and if not, the methods used to decide which results to collect. | 5 |
|  | 10b | List and define all other variables for which data were sought (e.g. participant and intervention characteristics, funding sources). Describe any assumptions made about any missing or unclear information. | 5 |
| Study risk of bias assessment | 11 | Specify the methods used to assess risk of bias in the included studies, including details of the tool(s) used, how many reviewers assessed each study and whether they worked independently, and if applicable, details of automation tools used in the process. | S1 |
| Effect measures | 12 | Specify for each outcome the effect measure(s) (e.g. risk ratio, mean difference) used in the synthesis or presentation of results. | 5 |
| Synthesis methods | 13a | Describe the processes used to decide which studies were eligible for each synthesis (e.g. tabulating the study intervention characteristics and comparing against the planned groups for each synthesis (item #5)). | 5 |
|  | 13b | Describe any methods required to prepare the data for presentation or synthesis, such as handling of missing summary statistics, or data conversions. | 5 |
|  | 13c | Describe any methods used to tabulate or visually display results of individual studies and syntheses. | Table 1 |
|  | 13d | Describe any methods used to synthesize results and provide a rationale for the choice(s). If meta-analysis was performed, describe the model(s), method(s) to identify the presence and extent of statistical heterogeneity, and software package(s) used. | 5 |
|  | 13e | Describe any methods used to explore possible causes of heterogeneity among study results (e.g. subgroup analysis, meta-regression). | NA |
|  | 13f | Describe any sensitivity analyses conducted to assess robustness of the synthesized results. | NA |
| Reporting bias assessment | 14 | Describe any methods used to assess risk of bias due to missing results in a synthesis (arising from reporting biases). | NA |
| Certainty assessment | 15 | Describe any methods used to assess certainty (or confidence) in the body of evidence for an outcome. | NA |
| **RESULTS** | | |  |
| Study selection | 16a | Describe the results of the search and selection process, from the number of records identified in the search to the number of studies included in the review, ideally using a flow diagram. | 6 and Figure 2 |
|  | 16b | Cite studies that might appear to meet the inclusion criteria, but which were excluded, and explain why they were excluded. | NA |
| Study characteristics | 17 | Cite each included study and present its characteristics. | 6 |
| Risk of bias in studies | 18 | Present assessments of risk of bias for each included study. | Table S3-5 |
| Results of individual studies | 19 | For all outcomes, present, for each study: (a) summary statistics for each group (where appropriate) and (b) an effect estimate and its precision (e.g. confidence/credible interval), ideally using structured tables or plots. | Table 1 |
| Results of syntheses | 20a | For each synthesis, briefly summarise the characteristics and risk of bias among contributing studies. | 6-8 |
|  | 20b | Present results of all statistical syntheses conducted. If meta-analysis was done, present for each the summary estimate and its precision (e.g. confidence/credible interval) and measures of statistical heterogeneity. If comparing groups, describe the direction of the effect. | 6-8 |
|  | 20c | Present results of all investigations of possible causes of heterogeneity among study results. | 8-13 |
|  | 20d | Present results of all sensitivity analyses conducted to assess the robustness of the synthesized results. | NA |
| Reporting biases | 21 | Present assessments of risk of bias due to missing results (arising from reporting biases) for each synthesis assessed. | NA |
| Certainty of evidence | 22 | Present assessments of certainty (or confidence) in the body of evidence for each outcome assessed. | NA |
| **DISCUSSION** | | |  |
| Discussion | 23a | Provide a general interpretation of the results in the context of other evidence. | 8-13 |
|  | 23b | Discuss any limitations of the evidence included in the review. | 12 |
|  | 23c | Discuss any limitations of the review processes used. | 13 |
|  | 23d | Discuss implications of the results for practice, policy, and future research. | 13 |
| **OTHER INFORMATION** | | |  |
| Registration and protocol | 24a | Provide registration information for the review, including register name and registration number, or state that the review was not registered. | 5 |
|  | 24b | Indicate where the review protocol can be accessed, or state that a protocol was not prepared. | 5 |
|  | 24c | Describe and explain any amendments to information provided at registration or in the protocol. | NA |
| Support | 25 | Describe sources of financial or non-financial support for the review, and the role of the funders or sponsors in the review. | 14 |
| Competing interests | 26 | Declare any competing interests of review authors. | 14 |
| Availability of data, code and other materials | 27 | Report which of the following are publicly available and where they can be found: template data collection forms; data extracted from included studies; data used for all analyses; analytic code; any other materials used in the review. | Table 1 |

Supplementary Table S2

*Specific Search Strings Used for PsycINFO, EMBASE, PubMed, Scopus, and Web of Science and the Associated Number of Search Results.*

| Database | Search terms | Number of Results |
| --- | --- | --- |
| PsycINFO | (("major depressive disorder" or depress* or MDD).ti. or ("major depressive disorder" or depress* or MDD).ab.) and ((emotion* or face or facial expression).ti. or (emotion* or face or facial expression).ab.) and (("effective connectivity" or "directional connectivity" or "Granger causality" or "dynamic causal modelling" or "dynamic causal modeling" or "structural equation modelling" or "structural equation modelling").ti. or ("effective connectivity" or "directional connectivity" or "Granger causality" or "dynamic causal modelling" or "dynamic causal modeling" or "structural equation modelling" or "structural equation modelling").ab.) | 165 |
| EMBASE | (("major depressive disorder" or depress* or MDD).ti. or ("major depressive disorder" or depress* or MDD).ab.) and ((emotion* or face or facial expression).ti. or (emotion* or face or facial expression).ab.) and (("effective connectivity" or "directional connectivity" or "Granger causality" or "dynamic causal modelling" or "dynamic causal modeling" or "structural equation modelling" or "structural equation modelling").ti. or ("effective connectivity" or "directional connectivity" or "Granger causality" or "dynamic causal modelling" or "dynamic causal modeling" or "structural equation modelling" or "structural equation modeling").ab.) | 320 |
| PubMed | ("major depressive disorder"[Title/Abstract] OR "depress*"[Title/Abstract] OR "MDD"[Title/Abstract]) AND ("emotion*"[Title/Abstract] OR "face"[Title/Abstract] OR "facial expression"[Title/Abstract]) AND ("effective connectivity"[Title/Abstract] OR "directional connectivity"[Title/Abstract] OR "Granger causality"[Title/Abstract] OR "dynamic causal modelling"[Title/Abstract] OR "dynamic causal modeling"[Title/Abstract] OR "structural equation modelling"[Title/Abstract] OR "structural equation modeling"[Title/Abstract]) | 294 |
| Scopus | (TITLE-ABS-KEY ( "major depressive disorder"  OR  depress*  OR  mdd )  AND  TITLE-ABS-KEY ( emotion*  OR  face  OR  facial  AND expression )  AND  TITLE-ABS-KEY ( "effective connectivity"  OR  "directional connectivity"  OR  "Granger causality"  OR  "dynamic causal modelling"  OR  "dynamic causal modeling"  OR  "structural equation modelling"  OR  "structural equation modeling" ) ) | 68 |
| Web of Science | (((TI=("major depressive disorder" or depress* or MDD) OR AB=("major depressive disorder" or depress* or MDD)) AND (TI=(emotion* or face or facial expression) OR AB=(emotion* or face or facial expression)) AND (TI=("effective connectivity" or "directional connectivity" or "Granger causality" or "dynamic causal modelling" or "dynamic causal modeling" or "structural equation modelling" or "structural equation modelling") OR AB=("effective connectivity" or "directional connectivity" or "Granger causality" or "dynamic causal modelling" or "dynamic causal modeling" or "structural equation modelling" or "structural equation modeling")))) | 294 |

Supplementary Table S3

*Quality Assessment Tool for Observational Cohort and Cross-Sectional Studies.*

|  | Q1 | Q2 | Q4 | Q5 | Q8 | Q9 | Q11 | Q12 | Q14 | Adjusted for | Rating  #1 | Rating  #2 |
| --- | --- | --- | --- | --- | --- | --- | --- | --- | --- | --- | --- | --- |
| de Almeida et al., 2009 | Y | Y | Y | N | Y | Y | Y | NA | N | - | Fair | Good |
| Carballedo et al., 2011 | Y | Y | Y | N | Y | Y | Y | NA | N | - | Fair | Good |
| de Almeida et al., 2011 | Y | Y | Y | N | Y | Y | Y | NA | N | - | Fair | Good |
| Tang et al., 2011 | Y | Y | Y | N | Y | Y | Y | NA | Y | Gender | Good | Good |
| Goulden et al., 2012 | Y | Y | Y | N | Y | Y | Y | NA | N | - | Good | Good |
| Lu et al., 2012 | Y | Y | Y | N | Y | Y | Y | NA | N | - | Fair | Good |
| Lu et al., 2013 | Y | Y | Y | N | Y | Y | Y | NA | N | - | Good | Good |
| Grant et al., 2014 | Y | Y | Y | N | Y | Y | Y | NA | N | - | Good | Good |
| Musgrove et al., 2015 | Y | Y | Y | N | Y | Y | Y | NA | N | - | Good | Good |
| Sacu et al., 2022 | Y | Y | Y | N | Y | Y | Y | NA | Y | Age, sex, education, study site and mean head motion | Good | Good |
| Willinger et al., 2022 | Y | Y | Y | N | Y | Y | Y | NA | Y | Age, sex, SSRI status, handedness | Good | Good |
| Li et al., 2022 | Y | Y | Y | N | Y | Y | Y | NA | N | - | Fair | Fair |

Y, Yes; N, No; NR, Not Reported; NA, Not Applicable; CD; Cannot Determine.

Q1. Was the research question or objective in this paper clearly stated?; Q2. Was the study population clearly specified and defined?; Q3. Was the participation rate of eligible persons at least 50%?; Q4. Were all the subjects selected or recruited from the same or similar populations (including the same time period)? Were inclusion and exclusion criteria for being in the study prespecified and applied uniformly to all participants?; Q5. Was a sample size justification, power description, or variance and effect estimates provided? Q6. For the analyses in this paper, were the exposure(s) of interest measured prior to the outcome(s) being measured? Q7. Was the timeframe sufficient so that one could reasonably expect to see an association between exposure and outcome if it existed? Q8. For exposures that can vary in amount or level, did the study examine different levels of the exposure as related to the outcome (e.g., categories of exposure, or exposure measured as continuous variable)? Q9. Were the exposure measures (independent variables) clearly defined, valid, reliable, and implemented consistently across all study participants? Q10. Was the exposure(s) assessed more than once over time? Q11. Were the outcome measures (dependent variables) clearly defined, valid, reliable, and implemented consistently across all study participants? Q12. Were the outcome assessors blinded to the exposure status of participants? Q13. Was loss to follow-up after baseline 20% or less? Q14. Were key potential confounding variables measured and adjusted statistically for their impact on the relationship between exposure(s) and outcome(s)?

Supplementary Table S4

*Quality Assessment of Controlled Intervention Studies.*

|  | Q1 | Q2 | Q3 | Q4 | Q5 | Q6 | Q7 | Q8 | Q9 | Q10 | Q11 | Q12 | Q13 | Q14 | Rating #1 | Rating  #2 |
| --- | --- | --- | --- | --- | --- | --- | --- | --- | --- | --- | --- | --- | --- | --- | --- | --- |
| Gilbert et al., 2021 | Y | NR | NR | Y | Y | Y | Y | Y | Y | Y | Y | NR | Y | Y | Fair | Fair |
| Jamieson et al., 2021 | Y | NR | NR | Y | Y | Y | Y | Y | NR | Y | Y | NR | Y | Y | Good | Good |

Y, Yes; N, No; NR, Not Reported; NA, Not Applicable; CD; Cannot Determine.

Q1. Was the study described as randomized, a randomized trial, a randomized clinical trial, or an RCT?; Q2. Was the method of randomization adequate (i.e., use of randomly generated assignment)?; Q3. Was the treatment allocation concealed (so that assignments could not be predicted)?; Q4. Were study participants and providers blinded to treatment group assignment?; Q5. Were the people assessing the outcomes blinded to the participants' group assignments?; Q6. Were the groups similar at baseline on important characteristics that could affect outcomes (e.g., demographics, risk factors, co-morbid conditions)?; Q7. Was the overall drop-out rate from the study at endpoint 20% or lower of the number allocated to treatment?; Q8. Was the differential drop-out rate (between treatment groups) at endpoint 15 percentage points or lower?; Q9. Was there high adherence to the intervention protocols for each treatment group?; Q10. Were other interventions avoided or similar in the groups (e.g., similar background treatments)?; Q11. Were outcomes assessed using valid and reliable measures, implemented consistently across all study participants?; Q12. Did the authors report that the sample size was sufficiently large to be able to detect a difference in the main outcome between groups with at least 80% power?; Q13. Were outcomes reported or subgroups analysed prespecified (i.e., identified before analyses were conducted)?; Q14. Were all randomized participants analysed in the group to which they were originally assigned, i.e., did they use an intention-to-treat analysis?

Supplementary Table S5

*Quality Assessment for Before-After (Pre-Post) Studies with No Control Group.*

|  | Q1 | Q2 | Q3 | Q4 | Q5 | Q6 | Q7 | Q8 | Q9 | Q10 | Q11 | Q12 | Rating #1 | Rating  #2 |
| --- | --- | --- | --- | --- | --- | --- | --- | --- | --- | --- | --- | --- | --- | --- |
| Vai et al., 2016 | Y | Y | Y | Y | CD | Y | Y | NR | Y | Y | N | NA | Good | Good |
| Kibleur et al., 2017 | Y | NR | N | CD | N | Y | Y | NA | Y | NR | N | NA | Fair | Fair |
| Frassle et al., 2020 | Y | Y | Y | Y | Y | N | Y | NA | Y | Y | NA | NA | Good | Fair |

Y, Yes; N, No; NR, Not Reported; NA, Not Applicable; CD; Cannot Determine.

Q1. Was the study question or objective clearly stated?; Q2. Were eligibility/selection criteria for the study population prespecified and clearly described?; Q3. Were the participants in the study representative of those who would be eligible for the test/service/intervention in the general or clinical population of interest?; Q4. Were all eligible participants that met the prespecified entry criteria enrolled?; Q5. Was the sample size sufficiently large to provide confidence in the findings?; Q6. Was the test/service/intervention clearly described and delivered consistently across the study population?; Q7. Were the outcome measures prespecified, clearly defined, valid, reliable, and assessed consistently across all study participants?; Q8. Were the people assessing the outcomes blinded to the participants' exposures/interventions?; Q9. Was the loss to follow-up after baseline 20% or less? Were those lost to follow-up accounted for in the analysis?; Q10. Did the statistical methods examine changes in outcome measures from before to after the intervention? Were statistical tests done that provided p values for the pre-to-post changes?; Q11. Were outcome measures of interest taken multiple times before the intervention and multiple times after the intervention (i.e., did they use an interrupted time-series design)?; Q12. If the intervention was conducted at a group level (e.g., a whole hospital, a community, etc.) did the statistical analysis take into account the use of individual-level data to determine effects at the group level?

Supplementary Table S6

*MRI Research Quality Assessment Tool Based on the Committee on Best Practices in Data Analysis and Sharing* *Guidelines*

| Category | Description | Score |
| --- | --- | --- |
| Experimental design | Stimuli used, number of block or trials, timing and duration, and presentation software | /4 |
| Acquisition reporting | Scanner (model, field strength & Tesla), coil, repetition time, pulse and spin/gradient echo frequency, and slice thickness reported | /5 |
| Preprocessing | Software, version, protocol described (e.g. realignment, slice-time correction, coregistration, normalization and spatial smoothing) | /3 |
| Statistical modelling and inference | ROIs used, how ROI were determined, modelling type used (DCM, Granger causality, or SEM), total model space/model structure detailed, and whether relevant covariates were used | /5 |
| Reporting of results | Effects in groups separately/overall, between group differences, and how between group effects were modelled | /3 |

Supplementary Table S7

*MRI Research Quality Assessment Tool*

| Study | Score / 20 |
| --- | --- |
| Willinger et al., 2022 | 20 |
| Jamieson et al., 2021 | 20 |
| Sacu et al., 2022 | 18 |
| Musgrove et al., 2015 | 17 |
| Vai et al., 2016 | 17 |
| Frässle et al., 2020 | 17 |
| Carballedo et al., 2011 | 15 |
| de Almeida et al., 2011 | 15 |
| Goulden et al., 2012 | 15 |
| de Almeida et al., 2009 | 14 |
| Grant et al., 2014 | 14 |

References

1. Study Quality Assessment Tools. <https://www.nhlbi.nih.gov/health-topics/study-quality-assessment-tools>, 2014, Accessed Date Accessed 2014 Accessed.

2. Davies G, Hayward M, Evans S, Mason O. A systematic review of structural MRI investigations within borderline personality disorder: Identification of key psychological variables of interest going forward. *Psychiat Res* 2020; **286**.

3. Nichols TE, Das S, Eickhoff SB, Evans AC, Glatard T, Hanke M *et al.* Best practices in data analysis and sharing in neuroimaging using MRI. *Nature Neuroscience* 2017; **20**(3)**:** 299-303.
